# Mechanisms of action and biocontrol potential of *Trichoderma* against *Alternaria* in horticultural crops: a review

**DOI:** 10.3389/ffunb.2026.1851718

**Published:** 2026-06-26

**Authors:** Rafi Khalido, Nia Rossiana, Dedat Prismantoro, Thomas Argyarich Jefferson, Malvin Albert, Nur Syafikah Abdullah, Budi Irawan, Mia Miranti, Wiwiek Harsonowati, Ravindra Chandra Joshi, Febri Doni

**Affiliations:** 1 Department of Biology, Faculty of Mathematics and Natural Sciences, Universitas Padjadjaran, Jatinangor, West Java, Indonesia; 2 Doctorate Program in Biotechnology, Graduate School, Universitas Padjadjaran, Bandung, West Java, Indonesia; 3 Centre for Research in Biotechnology for Agriculture (CEBAR), Universiti Malaya, Kuala Lumpur, Malaysia; 4 Research Center for Horticulture, National Research and Innovation Agency (BRIN), Cibinong, West Java, Indonesia; 5 Philippine Rice Research Institute, Maligaya, Science City of Muñoz, Muñoz, Nueva Ecija, Philippines

**Keywords:** *Alternaria*, biological control, fungal plant pathogens, horticultural crops, induced systemic resistance, *Trichoderma*

## Abstract

Fungal diseases caused by *Alternaria* species are major constraints to the productivity and quality of horticultural crops worldwide, resulting in significant yield losses, quality degradation, and postharvest challenges. Conventional management primarily relies on chemical fungicides; however, their extensive use raises concerns related to environmental contamination, pathogen resistance, and risks to human health. Consequently, increasing attention has shifted toward environmentally sustainable approaches such as microbial biological control agents. Among these, *Trichoderma* spp. have emerged as promising eco-friendly biocontrol agents due to their multifunctional interactions with plants and soil microbiomes. This review synthesizes current knowledge on the role of *Trichoderma* spp. in managing *Alternaria*-caused diseases in horticultural crops. Specific emphasis is placed on key mechanisms, including mycoparasitism, antibiosis through secondary metabolites, competition for nutrients and space, and the induction of systemic resistance in host plants. Evidence from *in vitro*, greenhouse, and field studies demonstrates that *Trichoderma* spp. can effectively suppress *Alternaria* pathogens while simultaneously enhancing plant growth and stress tolerance. However, the effectiveness of *Trichoderma*-based biocontrol strategies is influenced by environmental conditions, strain specificity, formulation stability, and interactions with indigenous microbial communities. Future research should focus on optimizing strain selection, developing robust formulations and delivery methods, and applying advanced genomic and metabolomic approaches to enhance the consistency and effectiveness of *Trichoderma*-based biocontrol in sustainable horticultural disease management.

## 1 Introduction

Horticultural crops, including various fruits and vegetables, play a central role in global food and nutritional security. They serve as primary sources of essential micronutrients, vitamins, dietary fiber, and health-promoting phytochemicals that are frequently deficient in diets dominated by staple grains (Ahmed et al., 2024a, 2024b; Al-Salim et al., 2025). Recent reviews emphasize that the sustained production and adequate consumption of these crops are fundamental for improving dietary diversity and nutritional quality (Ahmed et al., 2024a; Kathi et al., 2024; Creedon et al., 2025). Furthermore, regular intake of fiber-rich and phytochemical-rich produce promotes balanced dietary patterns, aligning with the priorities of contemporary food systems under pressure from population growth and shifting demands (Ahmed et al., 2024a; Kathi et al., 2024; Creedon et al., 2025).

In addition to their nutritional benefits, horticultural crops significantly promote agricultural economies by generating high-value produce and supporting employment throughout the value chain. They enhance household incomes and drive export earnings, particularly within smallholder-based production systems (Fikadu et al., 2025; Widadie et al., 2024; Jaenicke and Virchow, 2018). However, this high economic and nutritional value renders these crops highly susceptible to biotic stresses (Ali et al., 2023; Prechsl et al., 2023; Kumar and Kumar, 2024). Among these threats, fungal diseases represent major constraints to productivity and quality. In particular, *Alternaria* species are increasingly recognized as widespread and economically damaging pathogens that affect a diverse range of horticultural hosts globally (Ali et al., 2023; Mehmood et al., 2025; Deka et al., 2024).

Pathogens within the *Alternaria* genus are responsible for devastating conditions such as leaf blight and early blight, leading to significant reductions in both yield and marketable quality (Prechsl et al., 2023; Yessimseitova et al., 2025; Salotti et al., 2024). While yield losses can be severe depending on the crop type and environmental conditions, *Alternaria* infections also compromise postharvest storage and food safety through the production of harmful mycotoxins (Prechsl et al., 2023; Yessimseitova et al., 2025; Salotti et al., 2024). This economic burden is further amplified by cosmetic defects that diminish consumer acceptability and market value, often resulting in produce being rejected or sold at lower prices (Miranda-Apodaca et al., 2023; Slathia et al., 2021; Wang et al., 2025). In tropical and subtropical regions where environmental conditions favor pathogen development, the financial losses attributable to *Alternaria* are substantial, requiring effective management strategies (Miranda-Apodaca et al., 2023; Slathia et al., 2021; Wang et al., 2025).

Current conventional disease management strategies exhibit several limitations that undermine their long-term effectiveness (Beg et al., 2025; Garmendia et al., 2025). Chemical fungicides remain the primary approach, yet their frequent application raises concerns regarding environmental contamination, the development of pathogen resistance, and health risks to consumers and workers (Sharma et al., 2024). While cultural practices like crop rotation and sanitation can reduce disease incidence, their efficacy is often inconsistent under high disease pressure or in resource-limited regions (Ali et al., 2023; Prechsl et al., 2023). Moreover, reliance on a single management approach frequently fails to address the complex biology of *Alternaria* species, which can survive in soil, seed, and crop debris to enable rapid reinfection (Salotti et al., 2024; Erdogan and Guzel, 2025). These challenges underscore the urgent need for integrated, sustainable management strategies that protect environmental health and economic viability (Deka et al., 2024; Rasiukevičiūtė et al., 2025).

The limitations of synthetic chemicals have spurred growing interest in biological control agents (BCAs) as sustainable alternatives (Lahlali et al., 2022; Ons et al., 2020; Jefferson et al., 2026a). Unlike single-target fungicides, BCAs suppress phytopathogens through multifaceted mechanisms such as antagonism, competition, and parasitism (Nguyen et al., 2025; Köhl et al., 2019). These diverse modes of action enhance disease suppression while minimizing the risk of the pathogen developing resistance. Advances in microbial screening and formulation technologies have further improved the stability and field applicability of these biological agents (Tyagi et al., 2024; Villavicencio-Vásquez et al., 2025). Additionally, rising consumer demand for residue-free produce and stricter pesticide regulations have reinforced the relevance of biocontrol within modern horticultural systems (Chikte et al., 2024).

*Trichoderma* spp. are rhizosphere-associated filamentous fungi that have emerged as among the most extensively studied BCAs due to their ability to suppress a broad range of pathogens and enhance plant health (Guzmán-Guzmán et al., 2024; Raju and Punamalai, 2025; Jefferson et al., 2026b). These fungi are prevalent in the rhizosphere and endosphere, where they interact with plant roots and surrounding microbiomes to promote growth and suppress disease (Guzmán-Guzmán et al., 2024; Santillan-Culquimboz et al., 2025). Their efficacy is rooted in multifaceted modes of action, including direct mycoparasitism, nutrient competition, antibiosis via secondary metabolites, and the induction of systemic resistance in host plants (Guzmán-Guzmán et al., 2024; Gutiérrez-Moreno et al., 2025; Jefferson et al., 2026b). Furthermore, *Trichoderma* spp. act as plant growth-promoting fungi by stimulating nutrient uptake and enhancing resilience to abiotic stress (Chen et al., 2025; Raju and Punamalai, 2025).

The ecological adaptability of *Trichoderma* spp., including their ability to colonize diverse niches and modulate microbial communities, makes them a pivotal tool for reducing dependence on chemical pesticides (Guzmán-Guzmán et al., 2024; Raju and Punamalai, 2025). Consequently, *Trichoderma*-based approaches are increasingly regarded as core components of environmentally friendly disease management in horticultural production. This review aims to critically synthesize recent scientific evidence regarding the role of *Trichoderma* spp. in managing *Alternaria* diseases. Specifically, we consolidate current knowledge on the biological characteristics of *Trichoderma* spp., their antagonistic mechanisms against *Alternaria* species, and their effectiveness in both experimental and field settings. Furthermore, this review evaluates the potential of these biocontrol strategies within integrated disease management frameworks and identifies existing research gaps to provide an evidence-based foundation for sustainable horticultural practices.

## 2 Biology and pathogenicity of *Alternaria*

*Alternaria* is a taxonomically complex and ecologically versatile genus of filamentous fungi within the phylum Ascomycota, class Dothideomycetes, order Pleosporales, and family Pleosporaceae. This genus encompasses numerous species with saprophytic, endophytic, and pathogenic lifestyles across a wide range of substrates, including soil, plant tissues, seeds, and agricultural products (Li et al., 2022a; DeMers, 2022; Thomma, 2003). The taxonomy of *Alternaria* remains challenging because of pronounced morphological plasticity and overlapping conidial characteristics, which complicate species delimitation based solely on phenotypic traits (Li et al., 2022a; Bao et al., 2025). Consequently, recent studies have increasingly emphasized molecular phylogenetic approaches. These methods employ multilocus datasets, including ITS, β-tubulin, TEF1-α, RPB2, GAPDH, and Alt-a1, to improve taxonomic resolution and accurately delineate species boundaries within the genus (Li et al., 2022a; Bao et al., 2025). Such molecular tools have revealed substantial cryptic diversity, highlighting the limitations of traditional morphology-based classification systems (Li et al., 2022a; Bao et al., 2025; DeMers, 2022).

### 2.1 Major pathogenic species and host range

The most common phytopathogenic species reported globally include *A. alternata*, *A. solani*, *A. brassicicola*, and *A. tenuissima*, many of which occur as species complexes or host-associated pathotypes with broad host ranges (Yessimseitova et al., 2025; Blagojević et al., 2020; Fernandes et al., 2023). These species are associated with economically important diseases such as tomato early blight and leaf spot diseases in various horticultural crops. For example, *A. alternata* and *A. tenuissima* are frequently linked to tomato diseases, whereas *A. brassicicola* and *A. alternata* are commonly reported in leaf spot diseases affecting crops such as horseradish (Yessimseitova et al., 2025; Blagojević et al., 2020; Fernandes et al., 2023). In addition, some species, particularly *A. alternata*, are increasingly recognized as cryptic species complexes in which genetically similar isolates may differ substantially in host range, virulence, and toxin production, highlighting the importance of multilocus phylogenetic analyses for accurate pathogen characterization (Li et al., 2022a; Ebadi and Ebadi, 2024; DeMers, 2022).

Among the *Alternaria* taxa, *A. solani* is widely recognized as the primary causal agent of early blight in solanaceous crops such as potato and tomato, where it typically produces necrotic leaf lesions with characteristic concentric rings (Ghalib et al., 2025; Schmey et al., 2024). Under favorable environmental conditions, its characteristic necrotic lesions expand and coalesce, leading to extensive foliar damage and significant yield losses (Ghalib et al., 2025; Schmey et al., 2024). In contrast, *A. alternata* is a predominantly small-spored species causing leaf spot and brown spot diseases. Recent multilocus phylogenetic studies have confirmed the existence of multiple genetic lineages within the *A. alternata* complex that vary significantly in symptom expression and host specificity (Balodi et al., 2024; Dučkena et al., 2024; Ebadi and Ebadi, 2024).

Additionally, species such as *A. tenuissima* contribute to disease complexes in pepper and eggplant. Several *Alternaria* strains, particularly those phylogenetically related to *A. tenuissima*, are capable of producing multiple mycotoxins including alternariol, alternariol monomethyl ether, altenuene, and tenuazonic acid (Lan et al., 2023; Habib et al., 2021; Daichi et al., 2025). The production of these secondary metabolites exacerbates host tissue damage and further complicates disease management due to both phytotoxic effects and food safety implications (Habib et al., 2021; Lan et al., 2023).

### 2.2 Infection process and disease cycle

*Alternaria* species are generally regarded as necrotrophic pathogens that rely on host cell death for nutrient acquisition (Fernandes et al., 2023; Salotti et al., 2024; Wang et al., 2022a). Following deposition on susceptible host surfaces, conidia germinate in the presence of free water or high relative humidity. The resulting germ tubes penetrate leaf tissues through stomata, wounds, or directly through the cuticle. Enzymatic degradation of the cuticular layer plays a crucial role in this initial penetration (Fernandes et al., 2023; Ma et al., 2019). Once established, the fungi secrete a wide array of cell wall-degrading enzymes (CWDEs) and phytotoxins that induce host cell necrosis, facilitate nutrient acquisition from dead tissues, and accelerate lesion expansion (Fernandes et al., 2023; Jones and Perez, 2023; Salotti et al., 2024; Ma et al., 2019).

**Figure 1:**
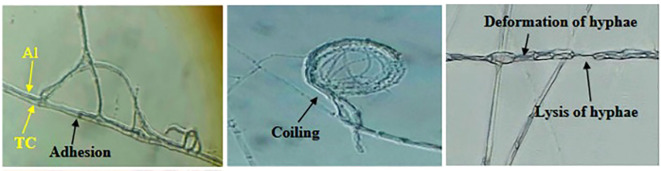
Microscopic stages of mycoparasitic interaction between *T. citrinoviride* (TC) and *Alternaria* sp. (Al). The micrographs illustrate the sequential stages of antagonistic interaction, including initial hyphal adhesion, coiling of *Trichoderma* around the host hyphae, followed by penetration of the pathogen cell wall, and subsequent lysis and deformation of *Alternaria* hyphae. These structural changes reflect cell wall degradation and cytoplasmic disintegration during the mycoparasitic process. All micrographs were obtained under light microscopy at 400× magnification. Source: Adapted from Al-Mekhlafi et al. (2025), licensed under CC BY-NC-ND 4.0.

The resulting necrotic lesions frequently serve as sources of secondary inoculum, supporting repeated cycles of conidial production and driving polycyclic disease development during the cropping season, as illustrated in **Figure 1**. Environmental factors such as leaf wetness duration, temperature, and host plant stress are critical in determining infection success and disease progression (Schmey et al., 2024). For instance, early blight caused by *A. solani* is typically favored by warm, humid conditions that enhance spore germination, penetration, and lesion development, allowing multiple infection cycles within a single growing season (Schmey et al., 2024; Fernandes et al., 2023; Jones and Perez, 2023; Salotti et al., 2024; Ma et al., 2019).

### 2.3 Mechanisms of virulence

The virulence of *Alternaria* spp. is determined by a combination of molecular and biochemical factors that enable successful host colonization, tissue destruction, and disease progression (Nagda and Meena, 2025; Cao et al., 2024; Jiang et al., 2023). Key determinants include the production of phytotoxic secondary metabolites, such as tenuazonic acid and alternariol, and the secretion of CWDEs including cellulases and pectinases. These factors often act synergistically to facilitate host tissue necrosis, suppress defense responses, and promote pathogen colonization, lesion expansion, and sporulation (Nagda and Meena, 2025; Zhan et al., 2025; Castaldi et al., 2023). Variation in the expression and composition of these virulence determinants among species and isolates contributes to differences in host range, disease severity, and epidemic potential. This variation is reflected in isolate-specific profiles of toxin production, cell wall–degrading enzyme activity, and genetic traits linked to pathogenic behavior (Nagda and Meena, 2025; Li et al., 2022b; Meena et al., 2017).

Many phytopathogenic species produce both host-specific toxins (HSTs) and non-host-specific toxins (nHSTs), which directly induce host cell death and disrupt cellular metabolism to contribute to tissue necrosis (Castaldi et al., 2023; Meena et al., 2017; Wang et al., 2022b). Species within the *A. alternata* complex synthesize a broad range of toxins, including alternariol, alternariol monomethyl ether, and tenuazonic acid, all of which are essential for symptom development and necrosis in susceptible hosts (Escrivá et al., 2017; Chen et al., 2021). Host-specific toxins function as primary virulence factors by targeting specific plant genotypes, thereby determining host susceptibility and influencing the outcome of plant–pathogen interactions (Cao et al., 2024; Meena et al., 2017; Tsuge et al., 2013). The diversity and regulation of toxin biosynthetic gene clusters among *Alternaria* strains generate a broad spectrum of secondary metabolites, complicating resistance breeding and disease management strategies due to strain-specific virulence profiles (Wang et al., 2022c; Kim and Dettman, 2025).

In addition to toxins, *Alternaria* spp. secrete a suite of CWDEs, including cellulases, pectinases, hemicellulases, and cutinases. These enzymes degrade plant cell walls, weaken host tissues, and facilitate penetration, tissue maceration, and nutrient acquisition (Ma et al., 2019; García-Calvo et al., 2018). Proteomic evidence demonstrates that these enzymes form a coordinated virulence arsenal that enhances fungal colonization and pathogenic success (Ma et al., 2019; Zhang et al., 2018). In necrotrophic pathotypes, the secretion of cutinases, cellulases, and pectate lyases is closely associated with lesion expansion, as mutants deficient in these enzymes exhibit significantly reduced lesion size (Ma et al., 2019). Comparative pathogenicity profiling further highlights a positive correlation between cell wall–degrading enzyme activity and virulence severity, confirming their role as essential determinants of pathogenicity in *Alternaria*–host interactions (Nagda and Meena, 2025).

### 2.4 Host-pathogen interactions

Interactions between *Alternaria* and their host plants represent a classical necrotrophic pathosystem in which disease progression relies on active host tissue destruction rather than prolonged colonization of living cells (Shao et al., 2025; Ma et al., 2019; Lawrence et al., 2008). Unlike biotrophic fungi that depend on living host tissues, *Alternaria* species exploit host cell death as a nutritional advantage and frequently convert plant defense-associated programmed cell death into susceptibility factors (Shao et al., 2025; Chung, 2012). This characteristic enables *Alternaria* to convert plant defense-associated cell death into a susceptibility factor, making necrosis an important component of disease progression rather than merely a symptom of infection (Mandal et al., 2019; Duhan et al., 2021; Xiao et al., 2024).

The infection process usually begins when fungal conidia adhere to the plant surface under favorable environmental conditions such as high humidity and moderate temperature (Fernandes et al., 2023; Salotti et al., 2024; Vinogradova et al., 2026). After attachment, the spores germinate and develop germ tubes and appressorium-like structures that facilitate penetration through natural openings such as stomata or through direct penetration of the cuticle and epidermal cell layers (Arya and Cohen, 2022; Fernandes et al., 2023). Following penetration, the fungus rapidly colonizes intercellular spaces and secretes various CWDEs, including cellulases, pectinases, xylanases, and cutinases, which degrade structural polysaccharides in plant tissues, weaken cell walls, and promote tissue maceration that facilitates fungal invasion and nutrient leakage (Ma et al., 2019; Rafiei et al., 2021; Fernandes et al., 2023).

A key determinant of *Alternaria* pathogenicity is the production of secondary metabolites and phytotoxins that directly damage host cells (Bacha et al., 2023; Salotti et al., 2024). Many *Alternaria* species produce host-selective toxins (HSTs), including AAL-toxin, ACT-toxin, and AM-toxin, which target susceptible host cultivars by disrupting membrane integrity, chloroplast function, mitochondrial metabolism, and sphingolipid biosynthesis (Tsuge et al., 2013; Meena and Samal, 2019; Wang et al., 2022c). These toxins induce severe physiological disturbances, leading to electrolyte leakage, membrane destabilization, chlorosis, and ultimately programmed cell death that creates nutrient-rich necrotic tissues favorable for fungal colonization (Tsuge et al., 2013; Meena and Samal, 2019; Wang et al., 2022b).

During infection, *Alternaria* also induces excessive accumulation of reactive oxygen species (ROS) such as hydrogen peroxide and superoxide radicals within host tissues (Meena and Samal, 2019; Munir et al., 2025). Although ROS are normally produced by plants as part of an early defense response against pathogens, necrotrophic fungi can exploit this oxidative burst to accelerate host cell death and tissue necrosis (Heller and Tudzynski, 2011; Newman and Derbyshire, 2020; Singh et al., 2021). High ROS accumulation promotes lipid peroxidation, protein oxidation, DNA damage, and loss of membrane integrity, ultimately resulting in cellular collapse that facilitates fungal expansion into surrounding tissues (Zhang et al., 2020; Nagda and Meena, 2026).

The interaction between *Alternaria* and host plants is further influenced by the regulation of plant immune signaling pathways (Liu et al., 2021, 2025; Munir et al., 2025). In many plant species, salicylic acid (SA)-mediated defense responses and hypersensitive response (HR)-associated localized cell death are highly effective against biotrophic pathogens but may inadvertently increase susceptibility to necrotrophic fungi such as *Alternaria* (Brouwer et al., 2020; Mishra et al., 2024). In contrast, jasmonic acid (JA)- and ethylene (ET)-mediated signaling pathways are generally associated with resistance against necrotrophic pathogens because they regulate defense genes involved in antimicrobial compound production, cell wall reinforcement, and detoxification processes (Gupta et al., 2020; Macioszek et al., 2023).

Host plants attempt to restrict *Alternaria* infection through multiple defense mechanisms, including the production of phytoalexins, antioxidant enzymes, pathogenesis-related (PR) proteins, and structural barriers such as lignification and callose deposition (Almagro et al., 2009; Wang et al., 2021). Antioxidant enzymes including catalase, peroxidase, and superoxide dismutase are activated to reduce oxidative stress caused by ROS accumulation during fungal invasion (Zhang et al., 2022a; Nagda and Meena, 2026). Collectively, *Alternaria*–host interactions represent a multilevel pathogenic system in which enzymatic penetration, toxin-mediated disruption, ROS-induced damage, immune modulation, and environmental factors act synergistically, with disease outcome ultimately determined by the dynamic interplay between fungal genetic potential, host resistance traits, and external conditions, highlighting the complexity of managing *Alternaria* diseases in agricultural systems.

## 3 Overview of *Trichoderma* as a biocontrol agent

*Trichoderma* is one of the most extensively studied genera of filamentous fungi in biological control. Its prominence is largely due to its taxonomic diversity, ecological plasticity, and functional versatility within agroecosystems (Woo et al., 2023; Kredics et al., 2024; Guzmán-Guzmán et al., 2024). Taxonomically, the genus belongs to the phylum Ascomycota, class Sordariomycetes, order Hypocreales, and family Hypocreaceae. It encompasses a large number of species that are predominantly free-living and fast-growing (Kubiak et al., 2023; Mukhopadhyay and Kumar, 2020). Members of *Trichoderma* are commonly encountered in soil, plant debris, and plant-associated niches, reflecting their strong saprophytic capacity and competitive fitness in microbially dense environments (Guzmán-Guzmán et al., 2024; Dutta et al., 2022; Rashmi et al., 2016).

### 3.1 Taxonomy and diversity

Historically, the taxonomy of *Trichoderma* was challenging due to the limited resolution of morphological characteristics, as traits such as conidial shape, colony morphology, and conidiophore branching often overlap among species and are strongly influenced by environmental conditions (Surma et al., 2025; Zheng et al., 2021). This limitation frequently led to misidentification and underestimation of species diversity within the genus. The introduction of molecular phylogenetic approaches, particularly multilocus sequence analysis (MLSA) based on ITS, TEF1-α, RPB2, and calmodulin genes, has significantly refined *Trichoderma* taxonomy by resolving cryptic species complexes and improving phylogenetic resolution within the genus (Arias Mota et al., 2025; Dou et al., 2020).

Based on current phylogenetic classification, *Trichoderma* belongs to the phylum Ascomycota, class Sordariomycetes, order Hypocreales, and family Hypocreaceae, and comprises a highly diverse genus with numerous phylogenetically distinct lineages. Within this framework, species such as *T. harzianum*, *T. asperellum*, *T. virens*, *T. atroviride*, and *T. koningii* represent well-characterized clades that are frequently recovered across different ecological niches, indicating substantial intra-genus diversity and ecological adaptability (Guzmán-Guzmán et al., 2023; Almeida et al., 2022). The clarification of these taxonomic relationships is essential for accurate species delimitation, as genetic differences among lineages are closely associated with ecological distribution, evolutionary divergence, and functional specialization.

### 3.2 Ecological adaptability

A key feature distinguishing *Trichoderma* spp. from many other microbial biocontrol agents is their remarkable ecological breadth and adaptability (Guzmán-Guzmán et al., 2024; Chen et al., 2025; Cavalcante et al., 2025). These fungi are ubiquitous in natural and agricultural environments, occurring in agricultural soils, forest litter, composts, decaying plant residues, and rhizospheres where they coexist and compete with a wide array of microorganisms (Guzmán-Guzmán et al., 2024; Dutta et al., 2022; Contreras-Cornejo et al., 2016). Their robust saprophytic lifestyle facilitates the efficient exploitation of organic substrates derived from plant residues. This allows *Trichoderma* spp. to compete effectively for limited nutrients in densely colonized soil environments and to maintain ecological dominance under competitive conditions (Contreras-Soto et al., 2025; Singh et al., 2024).

Their tolerance to environmental variability further supports the ecological success of *Trichoderma* spp. (Cavalcante et al., 2025). For example, Cavalcante et al. (2025) demonstrated that *Trichoderma* isolates can sustain growth across a pH range of 5 to 9 and exhibit substantial developmental plasticity beyond standard laboratory temperature regimes when adequate moisture is available. Furthermore, heat-treated strains of *T. harzianum* have been shown to remain viable under extreme pH conditions ranging from 3 to 12 and temperatures up to 45 °C, underscoring physiological adaptations that enable functionality under dynamic environmental conditions (Ahmed et al., 2025). This adaptability allows *Trichoderma* populations to survive agricultural disturbances such as tillage, irrigation, and fertilizer applications.

### 3.3 Colonization and persistence

Successful biological control depends not only on the antagonistic potential of a biocontrol agent but also on its ability to colonize and persist in plant-associated niches under diverse conditions (Riseh et al., 2025; Villavicencio-Vásquez et al., 2025). Additionally, *Trichoderma* spp. exhibit exceptional competence as root colonizers by establishing themselves in the rhizosphere and rhizoplane, where chemotropic responses to root exudates drive directed hyphal growth toward root surfaces and rapid proliferation along epidermal tissues (Taylor et al., 2022; Moreno-Ruiz et al., 2020). Through rapidly establishing strong associations within the rhizosphere and at potential pathogen entry points, *Trichoderma* spp. Promote early suppression of invading pathogens through niche occupation and competitive exclusion (Contreras-Cornejo et al., 2016; Dutta et al., 2023). This effective colonization strategy further restricts the availability of nutrients and physical space required for pathogen germination and establishment, thereby reducing the likelihood of successful *Alternaria* infection in plant tissues.

Root colonization typically persists throughout the growing season, maintaining interactions with both the host plant and the rhizosphere microbiota to enhance antagonistic activity and plant defense (Guzmán-Guzmán et al., 2024; Stummer et al., 2024). Certain strains can also colonize aboveground tissues, including leaves and stems, particularly following foliar application. This expands their functional range and reinforces their role in integrated disease management for horticultural crops (Şesan et al., 2020; Csótó et al., 2024). The persistence of *Trichoderma* populations is further supported by their ability to adapt to fluctuating rhizosphere conditions, interact dynamically with resident microbial communities, and maintain metabolic activity under environmental stress (Contreras-Cornejo et al., 2016; Shahriar et al., 2022; Guzmán-Guzmán et al., 2024). In some cases, *Trichoderma* spp. are also capable of forming biofilm-like structures on root surfaces, which improve adhesion, ecological stability, and long-term persistence in plant-associated environments (Triveni et al., 2013; Contreras-Cornejo et al., 2016; Taylor et al., 2022).

Sustained colonization is particularly important for effective suppression of *Alternaria* spp., as prolonged occupancy of plant-associated niches enables continuous antagonistic activity and early interception of invading pathogens before extensive infection can occur (Mukherjee et al., 2012; Coppola et al., 2019; Tyśkiewicz et al., 2022). Moreover, persistent interactions between *Trichoderma*, the host plant, and the surrounding microbiota contribute to the maintenance of plant defense readiness and overall ecological competitiveness within the rhizosphere (Tyśkiewicz et al., 2022; Dutta et al., 2023);. These characteristics highlight that successful biocontrol is not solely dependent on direct antagonism, but also on the capacity of *Trichoderma* spp. to establish stable and resilient populations under field conditions, thereby supporting more consistent and durable disease management outcomes.

## 4 Mechanisms of action of *Trichoderma* against *Alternaria*

Mycoparasitism is one of the best-characterized direct antagonistic mechanisms used by *Trichoderma* spp. against *Alternaria* spp (Philip et al., 2024; Guzmán-Guzmán et al., 2024; Nazla et al., 2026). Among the diverse antagonistic strategies employed by *Trichoderma* spp., including antibiosis through secondary metabolite and volatile organic compound production, competition for nutrients and ecological niches, and induction of plant systemic resistance, mycoparasitism is the most extensively studied mechanism at the molecular and cellular levels (Guzmán-Guzmán et al., 2024; Kredics et al., 2024; Chen et al., 2025). This process involves an active biological interaction in which *Trichoderma* spp. detect, physically engage, and degrade the pathogen’s hyphal structures through the secretion of hydrolytic enzymes such as chitinases, glucanases, and proteases that break down structural components of the pathogen cell wall (Dutta et al., 2023; Tyśkiewicz et al., 2022; Algifari et al., 2025). Unlike indirect suppression mechanisms, mycoparasitism requires direct physical contact between the biocontrol agent and the target pathogen (Stange et al., 2024a). Consequently, its effectiveness is strongly influenced by strain compatibility, environmental conditions, and spatial distribution within the plant–soil system (Smolińska and Kowalska, 2018; Rahman et al., 2024; El-Sheikh and Shahein, 2025).

In practice, these mechanisms rarely operate in isolation, as individual *Trichoderma* strains may suppress *Alternaria* pathogens through a combination of direct antagonism, metabolite production, resource competition, and host defense activation. Representative examples of these strain-specific interactions are summarized in **Table 1**, providing a concise overview of the main biocontrol mechanisms, target *Alternaria* species, host pathosystems, and reported mechanistic evidence.

**Table 1:** Representative examples of *Trichoderma* species/strains suppressing *Alternaria* pathogens through different biocontrol mechanisms.

Main biocontrol mechanisms	Target *Alternaria* species	Host plants/diseases	*Trichoderma* species/strains	Specific evidence/key findings	References
Mycoparasitism	*Alternaria* sp.	Horticultural crop/leaf spot or blight	*T. citrinoviride*	• Caused hyphal deformation and cell wall degradation in *Alternaria* sp. • Inhibited radial growth by 57.14%; metabolites provided additional inhibition. • Activated MAPK signaling and CWDE-related genes before direct contact.	Al-Mekhlafi et al. (2025); Abbas et al. (2022); Stange et al. (2024b)
Antibiosis/secondary metabolites	*A. alternata*	Tomato/leaf spot disease; postharvest pear/black spot disease	*T. afroharzianum* TRI07; *T. asperellum* 2-X-3-3; *T. hamatum* Ham34	• CWDEs, VOCs, and hyphal interactions disrupted pathogen growth and structure. • VOCs from *T. asperellum* inhibited growth by 74.4% and suppressed sporulation. • Peptaibols induced membrane leakage and fungal death.	Philip et al. (2024); Wu et al. (2025); Bjørk et al. (2022)
Competition for space and nutrients	*A. porri*	Horticultural crops; onion/purple blotch	*T. asperellum* ORF 2	• Rapid colonization and nutrient competition reduced pathogen growth. • Siderophore-mediated iron sequestration limited nutrient availability for *Alternaria*. • SEM showed hyphal intertwining and conidial damage.	Yao et al. (2023); Awal et al. (2025); Tyśkiewicz et al. (2022); Saini et al. (2025)
Induced systemic resistance (ISR)	*A. rassicicola*	Tomato or other horticultural crops; *Arabidopsis*/black leaf spot	*T. atroviride* ATCC 20476	• Activated JA/ET-related defenses and PR proteins. • Primed faster plant immune responses against *Alternaria*. • ISR involved *Trichoderma*-specific genes and additional SA-related signaling pathways.	Islam et al. (2023); Charpe et al. (2025); Sakai et al. (2024); Roychowdhury et al. (2024)
Plant growth promotion/stress resilience	*Alternaria* spp.; *A. alternata*	Horticultural crops; lettuce/leaf spot disease	*T. asperellum*	• Improved root growth, nutrient uptake, antioxidant activity, and stress tolerance. • Reduced oxidative stress and host susceptibility to *Alternaria* infection. • Genomic and metabolomic approaches are being used to improve biocontrol traits.	Chen et al. (2025); Basak et al. (2026); Metwally et al. (2026); Raju and Punamalai (2025)

### 4.1 Recognition and chemotropic growth

The initiation of mycoparasitism relies on *Trichoderma*’s capacity to perceive chemical and physical signals from prey fungi, such as *Alternaria*, which induce enzyme production and gene expression even before direct physical interaction occurs (Guzmán-Guzmán et al., 2023; Stange et al., 2024a). Diffusible molecules released from *Alternaria*, including cell wall fragments and low-molecular-weight metabolites, act as signals that stimulate the chemotropic growth of *Trichoderma* spp. toward the pathogen (Stange et al., 2024b). This directional growth enables *Trichoderma* spp. to locate their fungal host efficiently in competitive microbial environments (Stange et al., 2024a).

Moreno-Ruiz et al. (2020) demonstrated that *T. atroviride* can sense prey-derived diffusible compounds in fungal culture supernatants and respond through chemotropically guided hyphal growth. However, this recognition process is not uniform, as variability among *Alternaria* species and strain-specific sensing mechanisms in *Trichoderma* spp. result in differential antagonistic responses. At the molecular level, this recognition is mediated by surface-localized receptors that perceive extracellular cues (Abbas et al., 2022). Functional studies have shown that G-protein-coupled receptors, such as Gpr1, play central roles in sensing prey-derived signals and activating downstream signaling cascades associated with mycoparasitism (Omann et al., 2012; Abbas et al., 2022).

Upon activation, these pathways trigger regulatory networks involving mitogen-activated protein kinase (MAPK) cascades and calcium-dependent signaling (Atanasova et al., 2023). These networks coordinate the upregulation of genes encoding the cell wall–degrading enzymes, secondary metabolite biosynthetic pathways, and adhesion proteins (Vieira et al., 2013; Steindorff et al., 2014; Manzar et al., 2022). Philip et al. (2024) provided evidence that metabolites produced by *T. afroharzianum* TRI07 effectively inhibit the growth of *A. alternata*, confirming that these molecular mechanisms translate into tangible antagonistic activity.

### 4.2 Physical contact and hyphal coiling

Upon physical contact, *Trichoderma* spp. hyphae frequently coil around *Alternaria* hyphae, representing a morphological hallmark of mycoparasitism (Mukherjee et al., 2012; Mukhopadhyay and Kumar, 2020). This hallmark reflects coordinated recognition, attachment, and preparation for penetration driven by underlying signaling and enzymatic responses (Mukherjee et al., 2012; Mukhopadhyay and Kumar, 2020; Guzmán-Guzmán et al., 2023). Hyphal coiling likely enhances adhesion and stabilizes the host–parasite interaction, promoting the formation of a confined interface that enables the targeted secretion of enzymes (Sood et al., 2020; Tyśkiewicz et al., 2022).

Following adhesion, *Trichoderma* spp. develop penetration structures that facilitate the breaching of the *Alternaria* cell wall (Dutta et al., 2023; Santillan-Culquimboz et al., 2025). The efficiency of this penetration is strongly influenced by the structural and compositional features of the *Alternaria* cell wall, which vary among species such as *A. alternata* and *A. solani* (Fernandes et al., 2021; Ost et al., 2025). Although the core polysaccharide skeleton is conserved, the arrangement of beta-glucans, chitin, and glycoproteins varies, resulting in differences in mechanical rigidity (Hasim and Coleman, 2019; Zhao et al., 2023). Such structural variability helps explain the frequent discrepancy between strong *in vitro* antagonism and inconsistent disease suppression observed under field conditions (Whitaker and Bakker, 2019; Clough et al., 2022). However, the effectiveness of *Trichoderma* as a biocontrol agent is also influenced by multiple interacting factors, including environmental conditions, soil microbial interactions, and the ability of the fungus to establish and persist within plant-associated niches (Yao et al., 2023; Gao et al., 2022; Chen et al., 2025).

### 4.3 Enzymatic degradation and cellular collapse

The biochemical foundation of mycoparasitism lies in the production of lytic enzymes (Asad et al., 2015; Tyśkiewicz et al., 2022; Jefferson et al., 2026a). Chitinases hydrolyze chitin polymers, while glucanases target the glucan components that confer mechanical strength to the cell wall (Zhang et al., 2024a). Proteases further contribute by degrading structural proteins and facilitating membrane disruption (Deng et al., 2018; Silva et al., 2019; Abbas et al., 2022). As summarized in **Table 2**, CWDEs constitute important virulence factors in several *Alternaria* species, further emphasizing the significance of enzymatic degradation during *Trichoderma*-mediated antagonistic interactions.

**Table 2:** Overview of major *Alternaria* species, their host plants, disease symptoms, key toxins or secondary metabolites, and principal virulence factors.

*Alternaria* species	Major host plants	Disease symptoms	Major toxins/secondary metabolites	Key virulence factors	References
*A. Alternata*	Tomato, citrus, and various horticultural crops	Leaf spot; brown necrotic lesions with concentric rings, chlorotic halo, lesion coalescence, defoliation	Alternariol (AOH), alternariol monomethyl ether (AME), tenuazonic acid	CWDEs, host-specific toxins (HSTs), non-host-specific toxins (nHSTs)	Abdessemed et al., 2019; Chen et al., 2021; Fonseca-Guerra et al., 2023; Balodi et al., 2024; Ebadi and Ebadi, 2024
*A. solani*	Potato, tomato (Solanaceous crops)	Early blight; necrotic leaf lesions with concentric rings; lesion expansion and coalescence causing severe foliar damage	Alternaric acid, tenuazonic acid (TeA), alternariol (AOH), alternariol monomethyl ether (AME)	CWDEs, phytotoxins, necrotrophic infection strategy, enzymatic penetration	Xu et al., 2021; Fernandes et al., 2023; Ghalib et al., 2025; Schmey et al., 2024
*A. brassicicola*	Brassica crops, Horseradish (and other crops)	Leaf spot diseases; small black lesions with chlorotic halo, enlarging into concentric (target-like) spots, shot-hole symptoms	Brassicicolin A, tenuazonic acid (TeA), tentoxin	CWDEs, phytotoxins, host-specific toxins (HSTs), necrotrophic infection	Oka et al., 2005; Munir et al., 2020; Blagojević et al., 2020; Fernandes et al., 2023; Yessimseitova et al., 2025
*A. tenuissima*	Pepper, eggplant, various horticultural crops	Leaf spot; small brown necrotic spots, enlarging lesions, concentric rings (variable), coalescence, leaf senescence	Alternariol (AOH), alternariol monomethyl ether (AME), altenuene, tenuazonic acid	Mycotoxin production, CWDEs, necrotrophic strategy	Li et al., 2011; Habib et al., 2021; Lan et al., 2023; Fernandes et al., 2023; Daichi et al., 2025

Enzyme production is often inducible and is enhanced upon contact with *Alternaria*, reflecting an adaptive and energy-efficient strategy (Zeilinger and Omann, 2007; Rao et al., 2016). Variations in enzyme expression profiles among species and strains are linked to differences in biocontrol efficacy, highlighting the importance of precise strain selection (Saravanakumar et al., 2017; Garcés-Moncayo et al., 2025; Jefferson et al., 2026c). Moreover, environmental factors such as pH and nutrient availability can modulate enzyme activity (Kredics et al., 2004; Bao et al., 2024).

The combined effects of hyphal penetration and enzymatic degradation cause severe structural damage to *Alternaria* spp (Sood et al., 2020; Mukherjee et al., 2022; Yao et al., 2023). Microscopic analyses consistently demonstrate cell wall disintegration, cytoplasmic shrinkage, hyphal deformation, and eventual cellular collapse (Sood et al., 2020; Al-Mekhlafi et al., 2025). Al-Mekhlafi et al. (2025) provided representative microscopic evidence of these sequential stages, confirming the total degradation of pathogen hyphae during these antagonistic interactions.

### 4.4 Microscopic and quantitative evidence of antagonism

**Figure 2:**
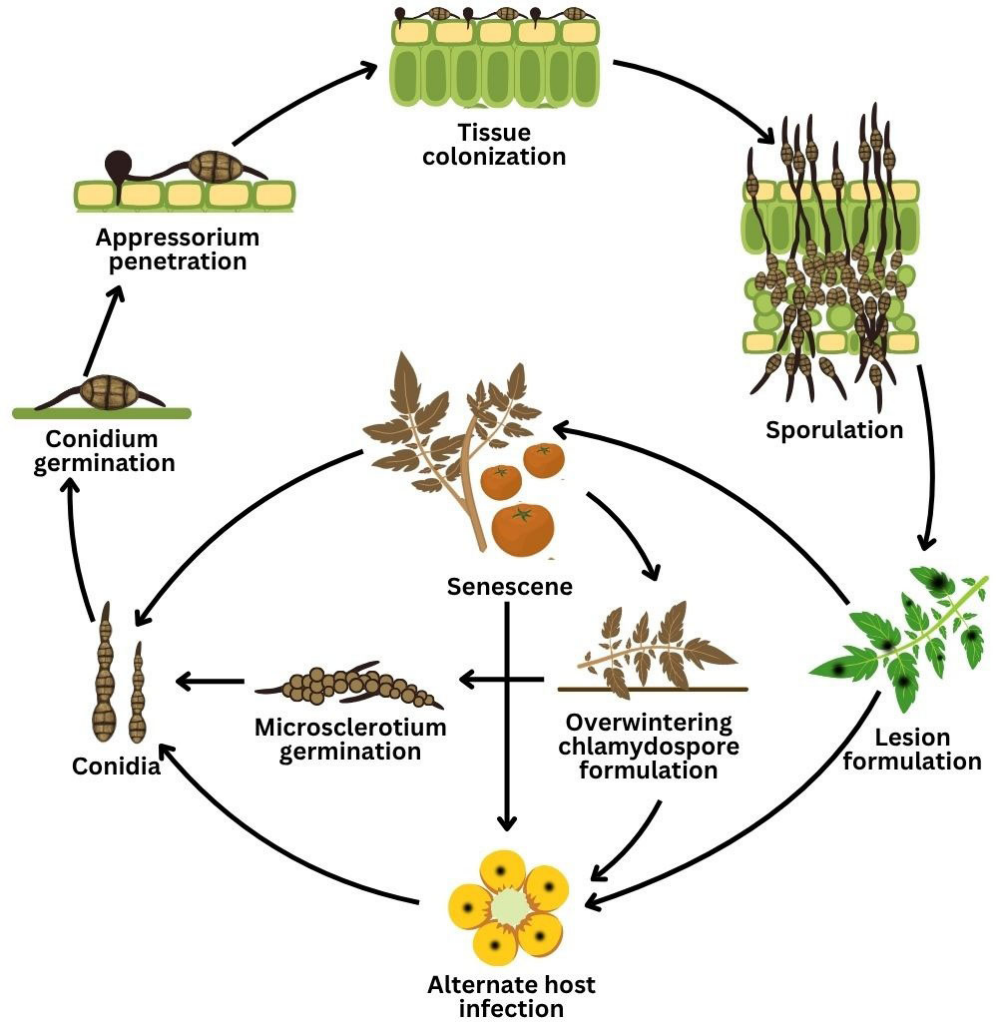
Overview of the infection cycle and mechanisms of *Alternaria* in horticultural crops, including conidial germination, appressorium-mediated penetration, tissue colonization, sporulation, lesion formation, and survival through overwintering structures.

Al-Mekhlafi et al. (2025) reported microscopic evidence confirming the strong mycoparasitic capacity of *T. citrinoviride* against *Alternaria* sp., as indicated by hyphal deformation, cell wall degradation, and cytoplasmic disintegration, as shown in **Figure 2**. These observations are consistent with mycoparasitic interactions involving direct hyphal contact (Dutta et al., 2023; Tyśkiewicz et al., 2022) and the enzymatic degradation of pathogen cell walls mediated by hydrolytic enzymes such as chitinases and glucanases (Algifari et al., 2025). Furthermore, *T. citrinoviride* inhibited the radial growth of *Alternaria* sp. by 57.14% in dual culture, while non-volatile and volatile metabolites contributed to additional inhibition of 48.21% and 36.36%, respectively (Al-Mekhlafi et al., 2025). Collectively, these findings indicate that antagonistic activity involves both direct interaction and metabolite-mediated effects.

While mycoparasitism represents a potent mechanism under laboratory conditions, its efficacy in field settings is often limited by environmental heterogeneity, microbial competition, and physical separation between organisms (Gutiérrez-Moreno et al., 2025; Ávila-Oviedo and Chávez-Avilés, 2026). Consequently, reliance on contact-dependent suppression alone may not provide stable disease control (Nguyen et al., 2025; Gutiérrez-Moreno et al., 2025; Ávila-Oviedo and Chávez-Avilés, 2026). Integrating mycoparasitism with antibiosis and induced systemic resistance is therefore essential for enhancing durability and ecological resilience in biocontrol systems.

### 4.5 Antibiosis and secondary metabolites

In addition to contact-dependent mycoparasitism, *Trichoderma* spp. exert antibiosis through the production of diverse secondary metabolites that inhibit pathogen growth (Guo et al., 2023; Ávila-Oviedo and Chávez-Avilés, 2026). These compounds include peptaibols, polyketides, terpenoids, gliotoxin, viridin, harzianolide, and 6-pentyl-α-pyrone (6-PP), many of which exhibit broad-spectrum antifungal activity (Flatschacher et al., 2025; Armenova et al., 2026). Unlike mechanical parasitism, antibiosis does not require direct hyphal contact, thereby extending the spatial and temporal scope of pathogen suppression (Arseneault and Filion, 2017).

Mechanistically, these metabolites interfere with fungal physiology at multiple levels, including membrane permeabilization, disruption of mitochondrial respiration, oxidative imbalance, and inhibition of cell wall biosynthesis (Khan et al., 2020; Chen et al., 2025; Ávila-Oviedo and Chávez-Avilés, 2026). Peptaibols, for instance, integrate into lipid bilayers to form ion channels, leading to cytoplasmic leakage, membrane depolarization, and eventual cell death (Zhang et al., 2022b; Bjørk et al., 2022). In contrast, several polyketides- and terpene-derived metabolites perturb intracellular redox homeostasis and interfere with signaling networks, leading to mitochondrial dysfunction and impaired morphogenesis (Elhamouly et al., 2022; Wadhwa et al., 2024; Angeles Flores et al., 2026). Collectively, these multifaceted modes of action underscore the capacity of *Trichoderma*-derived metabolites to exert systemic physiological disruption rather than localized damage.

In *Alternaria*, where pathogenicity depends on sustained hyphal expansion and regulated toxin biosynthesis, metabolic disruption likely compromises both vegetative growth and virulence factor production (Meena et al., 2017; You et al., 2023). Restricted ATP generation and precursor availability may impair signaling pathways that regulate secondary metabolite biosynthesis (Li et al., 2024a). Although species-specific metabolomic profiling of *Alternaria*–*Trichoderma* interactions remains limited, the conservation of central carbon metabolism and oxidative stress responses across filamentous fungi suggests a broad susceptibility to these bioactive compounds (Hong et al., 2013; Nielsen et al., 2019; Yaakoub et al., 2022).

### 4.6 Volatile and non-volatile inhibitory compounds

Antibiosis in *Trichoderma* spp. involves both diffusible (non-volatile) metabolites and volatile organic compounds (VOCs), each contributing to ecological fitness and pathogen suppression (Joo and Hussein, 2022; Chávez-Avilés et al., 2024). Non-volatile metabolites accumulate in the rhizosphere or infection microenvironment, where they exert concentration-dependent inhibitory effects on neighboring pathogens (Li et al., 2019; Rahman et al., 2023). In contrast, VOCs such as 6-PP and various sesquiterpenes can diffuse through air-filled soil pores, enabling inhibition without physical proximity (Gualtieri et al., 2022; Jiménez-Bremont et al., 2024).

The ecological significance of VOC-mediated inhibition depends on its capacity to overcome spatial constraints that may limit direct mycoparasitic contact (Morath et al., 2012; Guo et al., 2019; Stange et al., 2024b). In heterogeneous soil systems where microbial distribution is patchy, volatile-mediated signaling and inhibition may represent a more reliable suppression mechanism than contact-dependent interactions alone (Garbeva et al., 2014; Schulz-Bohm et al., 2015; Effmert et al., 2012). Moreover, VOCs have been reported to modulate fungal gene expression, including genes associated with sporulation and stress response, further amplifying their suppressive effects (Li et al., 2024a; Li et al., 2024b; Abdenaceur et al., 2024).

The antifungal activity of *Trichoderma*-derived metabolites is reflected in a range of physiological and structural alterations in target pathogens (Prapagdee et al., 2008; Inayati et al., 2019; Halo et al., 2018). Exposure to these compounds results in reduced mycelial growth, accompanied by morphological abnormalities such as hyphal swelling, distortion, and irregular branching (Prapagdee et al., 2008; Moya et al., 2018; Karličić et al., 2021). In addition, a marked decrease in conidial germination is commonly observed, indicating that both vegetative development and early infection stages are highly susceptible to metabolite-mediated inhibition (Rahnama et al., 2025; Xu et al., 2026).

At the cellular level, these effects are associated with significant disruptions in fungal integrity and metabolic balance (Saadaoui et al., 2023; Philip et al., 2024). Philip et al. (2024) demonstrated that antifungal metabolites produced by *T. afroharzianum* disrupt the structural integrity of *A. alternata* through the production of cell wall–degrading enzymes (e.g., chitinases and glucanases) and toxic volatile organic compounds, as well as through direct hyphal interactions such as coiling and penetration, ultimately inhibiting fungal growth and impairing normal physiological functions. Such alterations suggest that the inhibitory effects extend beyond surface-level structural damage, potentially interfering with essential cellular processes and contributing to the observed suppression of fungal growth and development (Khan et al., 2020; Zhang et al., 2024b).

### 4.7 Competition for space and nutrients

*Trichoderma* spp. effectively suppress pathogens through competition for space (Yao et al., 2023; Awal et al., 2025; Ávila-Oviedo and Chávez-Avilés, 2026). As demonstrated by Awal et al. (2025), where *T. yunnanense* TM10 inhibited pathogen growth via spatial competition. Their fast growth rate, high reproductive capacity, and adaptability to diverse environmental conditions enable them to occupy available niches more efficiently than many phytopathogenic fungi, including *Alternaria* (Halifu et al., 2019; Yao et al., 2023). This rapid colonization physically restricts pathogen access to infection sites and establishes an early competitive advantage, reducing the likelihood of successful pathogen adhesion.

Beyond spatial dominance, *Trichoderma* spp. actively compete for essential nutrients, particularly carbon, nitrogen, and micronutrients (Pozo et al., 2024; Villavicencio-Vásquez et al., 2025; Guzmán-Guzmán et al., 2024). Through efficient uptake systems and metabolic versatility, *Trichoderma* spp. can utilize a wide range of organic substrates, allowing them to thrive even under nutrient-limited conditions (Zin and Badaluddin, 2020; Santillan-Culquimboz et al., 2025; Ávila-Oviedo and Chávez-Avilés, 2026). This competitive nutrient acquisition deprives *Alternaria* of the critical resources necessary for spore germination, hyphal elongation, and colonization, thereby suppressing their growth and pathogenic potential.

Among micronutrients, iron plays a significant role in microbial competition. It is essential for various cellular processes but is often limited in bioavailable forms in the environment (Manzar et al., 2022; Cui et al., 2025; Santillan-Culquimboz et al., 2025). *Trichoderma* spp. produce siderophores, which are low molecular weight iron-chelating compounds that efficiently sequester iron (Moropana et al., 2024; Cui et al., 2025). Through binding iron with high affinity, siderophores reduce its availability to competing pathogens, effectively creating iron-depleted conditions that inhibit pathogen metabolism and growth. (Tyśkiewicz et al., 2022; Cui et al., 2025). This mechanism not only enhances the competitive fitness of *Trichoderma* spp. but also contributes significantly to their biocontrol efficacy against fungal pathogens such as *Alternaria* (Contreras-Cornejo et al., 2016; Khan et al., 2020; Cui et al., 2025). Collectively, the combined effects of rapid colonization, efficient nutrient acquisition, and siderophore-mediated iron competition highlight the importance of resource competition as a key mechanism in *Trichoderma*-mediated biocontrol.

### 4.8 Induced systemic resistance and plant defense

*Trichoderma* spp. enhance plant defense through indirect strategies, particularly by inducing systemic resistance (ISR) (Islam et al., 2023; Guzmán-Guzmán et al., 2024; Basak et al., 2026). Following root colonization, *Trichoderma* spp. interact with plant cells to activate key defense signaling pathways. These are primarily mediated by jasmonic acid (JA) and ethylene (ET), which are commonly associated with resistance against necrotrophic pathogens (Shoresh et al., 2005; Islam et al., 2023; Jung et al., 2024). Rather than causing strong stress responses, this interaction primes the plant immune system, enabling a faster and more effective defense upon subsequent pathogen attack (Islam et al., 2023; Hönig et al., 2023; Jung et al., 2024).

At the molecular level, *Trichoderma* spp. release elicitor molecules, including small, secreted proteins, secondary metabolites, and cell wall–derived fragments, which are recognized by plant receptors (Guzmán-Guzmán et al., 2024; Pereira et al., 2025; Chen et al., 2025). This recognition triggers a cascade of intracellular signaling events that activate systemic defense throughout the plant (Charpe et al., 2025). Consequently, plants exhibit an enhanced defensive state even in tissues distant from the initial colonization site, highlighting the importance of ISR in *Trichoderma*-mediated biocontrol.

This activation is closely linked to the transcriptional reprogramming of plant cells (Aerts et al., 2022; Khan et al., 2022). In response to *Trichoderma* spp. colonization, a wide range of defense-related genes are upregulated, including those encoding pathogenesis-related (PR) proteins, chitinases, β-1,3-glucanases, and other antimicrobial enzymes (Intana et al., 2022; Aamir et al., 2023; Shanmugaraj et al., 2024). These proteins play a direct role in degrading fungal cell walls and limiting pathogen establishment (Ribeiro et al., 2019; Safeer et al., 2025; Kumari et al., 2025). *Trichoderma*-induced priming ensures that these defense genes are expressed more rapidly and at higher levels upon pathogen challenge (Shanmugaraj et al., 2024; Chen et al., 2025; Basak et al., 2026). This allows plants to maintain a balance between growth and defense, avoiding unnecessary energy expenditure under non-stress conditions (Jatana et al., 2024; Basak et al., 2026; Dong and Ahammed, 2026). As a result, plants become more resistant to a broad spectrum of pathogens, including fungi such as *Alternaria* spp., as reported by Charpe et al. (2025), where microbial-mediated induced resistance enhances plant defense capacity and primes plants for “stronger and faster defense responses,” ultimately inhibiting pathogen growth and spore germination and rendering *Alternaria* spp. particularly sensitive to enhanced host defense responses.

### 4.9 Plant growth promotion and stress resilience

In parallel with defense induction, *Trichoderma* spp. significantly contribute to plant growth promotion by enhancing physiological performance and growth parameters such as root development and biomass accumulation (Doni et al., 2023; Chen et al., 2025; Jefferson et al., 2026a). Root colonization stimulates architecture development, enhances nutrient uptake efficiency, and improves overall plant vigor (Doni et al., 2014; Sood et al., 2020; Akbari et al., 2024). These effects are often associated with the production of growth-regulating compounds, including auxin-like phytohormones, as well as enhanced nutrient mobilization and availability in the rhizosphere (Fiorentino et al., 2018; Andrzejak and Janowska, 2022; Wang et al., 2024). In the context of *Alternaria* management, improved root architecture and enhanced nutrient status enable plants to mount faster and more robust defense responses upon pathogen challenge, as higher physiological vigor directly strengthens both constitutive and inducible resistance mechanisms (Gholizadeh Vazvani et al., 2026).

Under stress conditions, the presence of *Trichoderma* further enhances plant resilience by modulating biochemical responses (Zhang et al., 2016; Contreras-Cornejo et al., 2024; Geng et al., 2025). Plants treated with *Trichoderma* frequently exhibit improved tolerance to abiotic stresses such as drought, salinity, and oxidative stress, alongside increased resistance to infection (Contreras-Cornejo et al., 2024; Guzmán-Guzmán et al., 2024; Chen et al., 2025). This is particularly relevant in the context of *Alternaria* pathogenesis, as abiotic stress conditions are well-documented to predispose host plants to *Alternaria* infection by compromising cell wall integrity, suppressing antioxidant capacity, and downregulating defense-related gene expression (Kissoudis et al., 2014; Munir et al., 2025**).** *Trichoderma*-mediated stress priming therefore reduces host vulnerability by maintaining basal defense competence even under unfavorable environmental conditions, effectively narrowing the physiological window of susceptibility exploited by *Alternaria* spp. during infection (Pacheco-Trejo et al., 2022; Aamir et al., 2023; Basak et al., 2026). This dual functionality highlights the role of *Trichoderma* as an effective and sustainable agent not only in general plant health management, but specifically in reducing host susceptibility to *Alternaria*-associated diseases.

## 5 Biocontrol potential of *Trichoderma* in horticultural crops

### 5.1 *In vitro* evidence

Dual culture assays are among the most widely used and fundamental experimental approaches for evaluating the antagonistic potential of *Trichoderma* spp. against phytopathogenic fungi such as *Alternaria* spp (Naglot et al., 2015; Olowe et al., 2022; Nysanth et al., 2022). Under controlled laboratory conditions, this method enables direct observation of fungal–fungal interactions, including growth inhibition, spatial competition, and morphological responses at the interface of interaction (Nysanth et al., 2022; Dourou and La Porta, 2023; Ren et al., 2025). Typically, *Trichoderma* and the target pathogen are co-cultured on the same agar medium, allowing for the quantitative assessment of radial growth inhibition as well as the qualitative characterization of interaction dynamics (Stracquadanio et al., 2020; Go et al., 2023; Ren et al., 2025).

Numerous studies have demonstrated that *Trichoderma* spp. significantly inhibit the mycelial growth of *Alternaria* spp. in dual culture systems, often resulting in substantial reductions in colony expansion and competitive exclusion of the pathogen (Arzanlou et al., 2014; Yassin et al., 2022; Philip et al., 2024). For example, Yassin et al. (2022) demonstrated through dual culture assays that *T. viride* and *T. harzianum* significantly suppressed the growth of *A. alternata*, achieving inhibition levels of 75.04% and 67.83%, respectively, thereby demonstrating their strong antagonistic potential under *in vitro* conditions. Such inhibitory effects are typically quantified using the percentage inhibition of radial growth (PIRG), which is a widely adopted metric that enables standardized comparison of antagonistic performance across different strains and experimental conditions (Stracquadanio et al., 2020). In many cases, highly effective *Trichoderma* isolates are capable of rapidly colonizing the culture medium, overgrowing *Alternaria* colonies, and establishing physical dominance within a short incubation period (Abo-Elyousr et al., 2014; Gao et al., 2025; Al-Mekhlafi et al., 2025).

The effectiveness of *Trichoderma* spp. in dual culture systems is closely linked to strain-specific traits, including growth rate, enzyme production capacity, and secondary metabolite profiles (Kumari et al., 2024; Garcés-Moncayo et al., 2025; Chen et al., 2025). Accordingly, certain strains, particularly *T. harzianum* and *T. asperellum*, consistently exhibit strong antagonistic performance, achieving high levels of pathogen suppression under laboratory conditions, with inhibition frequently exceeding 70% depending on the strain and pathogen (Kuzmanovska et al., 2018; Gao et al., 2025; Atlagić et al., 2025). However, significant variability in inhibition efficiency among strains highlights the critical importance of isolate selection during biocontrol development (Yendyo et al., 2017; Kumari et al., 2024).

Despite their widespread use, dual culture assays have inherent limitations that must be considered when interpreting results (Liu et al., 2022; Kumari et al., 2024; Alsalmo et al., 2026). Laboratory conditions provide a simplified and homogeneous environment that does not fully reflect the complexity of soil ecosystems, plant–microbe interactions, or environmental variability encountered under field conditions (Moënne-Loccoz et al., 2014; Bardin et al., 2015). Consequently, strong *in vitro* antagonism does not always translate into consistent disease suppression in planta or under field conditions. This discrepancy underscores the need to integrate dual culture findings with greenhouse and field-based evaluations to achieve a more comprehensive and reliable assessment of biocontrol potential.

### 5.2 Greenhouse studies

Greenhouse experiments serve as an essential intermediate step between *in vitro* assays and field applications. They provide a semi-controlled environment that accurately reflects plant–microbe interactions while maintaining experimental reproducibility (Forero et al., 2019; Aznar-Sánchez et al., 2020; Slimani et al., 2023). Under these conditions, numerous studies have demonstrated that *Trichoderma* spp. significantly reduce the disease severity caused by *Alternaria* spp. across a range of horticultural crops (Khalil et al., 2021; Imran et al., 2023; Sharma et al., 2024). This disease suppression is typically associated with both direct antagonistic activity in the rhizosphere and the activation of plant defense mechanisms, particularly induced systemic resistance (Dutta et al., 2023; Yao et al., 2023; Kaman et al., 2024). Plants treated with *Trichoderma* often exhibit reduced lesion size, delayed symptom development, and lower disease incidence compared to untreated controls, indicating the effective suppression of pathogen establishment and progression (Tyśkiewicz et al., 2022; Mishu et al., 2025; Herrera-Rodriguez et al., 2026).

In addition to disease control, greenhouse studies consistently report the positive effects of *Trichoderma* spp. on plant growth parameters (Imran et al., 2023; Dutta et al., 2024; Philip et al., 2024). Notable improvements include enhanced root and shoot biomass, increased plant height, expanded leaf area, and greater chlorophyll content (Ban et al., 2018; Dutta et al., 2024; Philip et al., 2024). Such growth promotion is closely linked to improved nutrient uptake, the production of phytohormones like auxin-like compounds, and the modulation of plant physiological processes (Andrzejak and Janowska, 2022; Contreras-Cornejo et al., 2024; Chen et al., 2025). Importantly, the dual role of *Trichoderma* as both a biocontrol agent and a plant growth–promoting fungus highlights its potential to simultaneously enhance plant health and productivity under controlled yet biologically relevant conditions (Andrzejak and Janowska, 2022; Imran et al., 2023; Dutta et al., 2024).

### 5.3 Field applications

Field applications provide the most realistic evaluation of *Trichoderma*-based biocontrol strategies because they incorporate the full complexity of environmental variability, soil heterogeneity, and microbial interactions (Tyśkiewicz et al., 2022; Guzmán-Guzmán et al., 2024; Ávila-Oviedo and Chávez-Avilés, 2026). Several case studies in horticultural crops have demonstrated that *Trichoderma* spp. can effectively reduce the disease incidence caused by *Alternaria* spp., although the magnitude of suppression is often more variable than under controlled conditions (Metz and Hausladen, 2022; Imran et al., 2022, 2023). Successful field applications are generally associated with proper strain selection, appropriate formulation, and optimal timing of application. All of these factors influence colonization efficiency and persistence in the soil or on plant surfaces (dela Cruz et al., 2023; Chen et al., 2025; Villavicencio-Vásquez et al., 2025; Jefferson et al., 2026c).

Beyond disease suppression, field studies frequently report improvements in crop yield and quality following *Trichoderma* application (Dutta et al., 2024; García-Latorre et al., 2025; Herrera-Rodriguez et al., 2026). These benefits are attributed not only to reduced pathogen pressure but also to enhanced plant vigor and improved tolerance to abiotic and biotic stresses. These advantages are mediated by *Trichoderma* interactions with host plants (Awad-Allah et al., 2023; Chen et al., 2025; Geng et al., 2025). Yield increases are often accompanied by improved fruit size, uniformity, and marketability, which are critical parameters in horticultural production systems (Vultaggio et al., 2024; García-Latorre et al., 2025). However, variability in field performance remains a common observation, reflecting the influence of environmental and ecological factors that are absent in laboratory and greenhouse studies.

### 5.4 Factors influencing the efficacy of *Trichoderma*

The effectiveness of *Trichoderma* as a biocontrol agent is influenced by multiple interacting factors that determine its establishment, persistence, and functional activity in agroecosystems. These include environmental conditions, soil microbial interactions, and the ability of the fungus to colonize plant roots (Yao et al., 2023; Gao et al., 2022; Chen et al., 2025). Among these, environmental conditions such as temperature, soil moisture, pH, and nutrient availability play a critical role in shaping both *Trichoderma* performance and pathogen dynamics (Daryaei et al., 2016; Cavalcante et al., 2025; Gutiérrez-Moreno et al., 2025). Variations in these factors can significantly affect fungal growth, enzyme activity, and metabolite production, ultimately influencing disease suppression outcomes (Cortez-Lázaro et al., 2025; Algifari et al., 2025; Ávila-Oviedo and Chávez-Avilés, 2026).

Strain specificity is another key determinant of efficacy because different *Trichoderma* isolates exhibit distinct physiological traits, antagonistic capacities, and ecological adaptability (Jambhulkar et al., 2024; Baek et al., 2025; Garcés-Moncayo et al., 2025). These differences underscore the importance of selecting well-adapted and functionally efficient strains for specific crops and environments (Jambhulkar et al., 2024; Baek et al., 2025; Garcés-Moncayo et al., 2025). In addition, formulation and delivery methods, such as seed treatment, soil application, or foliar sprays, strongly influence the success of biocontrol by affecting the survival, distribution, and colonization of the biocontrol agent (Martinez et al., 2023; Vindas-Reyes et al., 2024).

Interactions with indigenous microbiota further complicate the performance of *Trichoderma* spp. under field conditions. Their efficacy can depend on interactions with the surrounding soil microbiome and associated microbial communities (Yao et al., 2023; Shao et al., 2025; Guzmán-Guzmán et al., 2024). Native microbial communities can either enhance or suppress the activity of introduced strains through competition, synergism, or niche exclusion (Yao et al., 2023; Shao et al., 2025). Consequently, the outcome of biocontrol applications depends not only on the intrinsic properties of *Trichoderma* spp. but also on the broader ecological context in which they are deployed.

## 6 Challenges and limitations

Despite its promising potential, the application of *Trichoderma* in horticultural systems faces several challenges that limit its consistent performance (Gutiérrez-Moreno et al., 2025; Llanos-Gómez et al., 2026). One of the primary constraints is the variability of field efficacy, as environmental fluctuations and complex microbial interactions can lead to inconsistent disease suppression compared to controlled conditions (Bandara and Kang, 2024; Gutiérrez-Moreno et al., 2025). This limitation is particularly evident in mycoparasitism, which, although highly effective under laboratory conditions, often shows reduced efficacy in field environments due to environmental heterogeneity, microbial competition, and physical separation between organisms (Gutiérrez-Moreno et al., 2025; Ávila-Oviedo and Chávez-Avilés, 2026). As a result, reliance solely on contact-dependent suppression mechanisms may not ensure stable and consistent disease control under diverse agricultural conditions (Nguyen et al., 2025; Gutiérrez-Moreno et al., 2025; Ávila-Oviedo and Chávez-Avilés, 2026). Therefore, integrating mycoparasitism with complementary mechanisms such as antibiosis and induced systemic resistance is considered essential for improving the durability, consistency, and ecological resilience of *Trichoderma*-based biocontrol systems, particularly across different environmental conditions and growing seasons.

**Figure 3:**
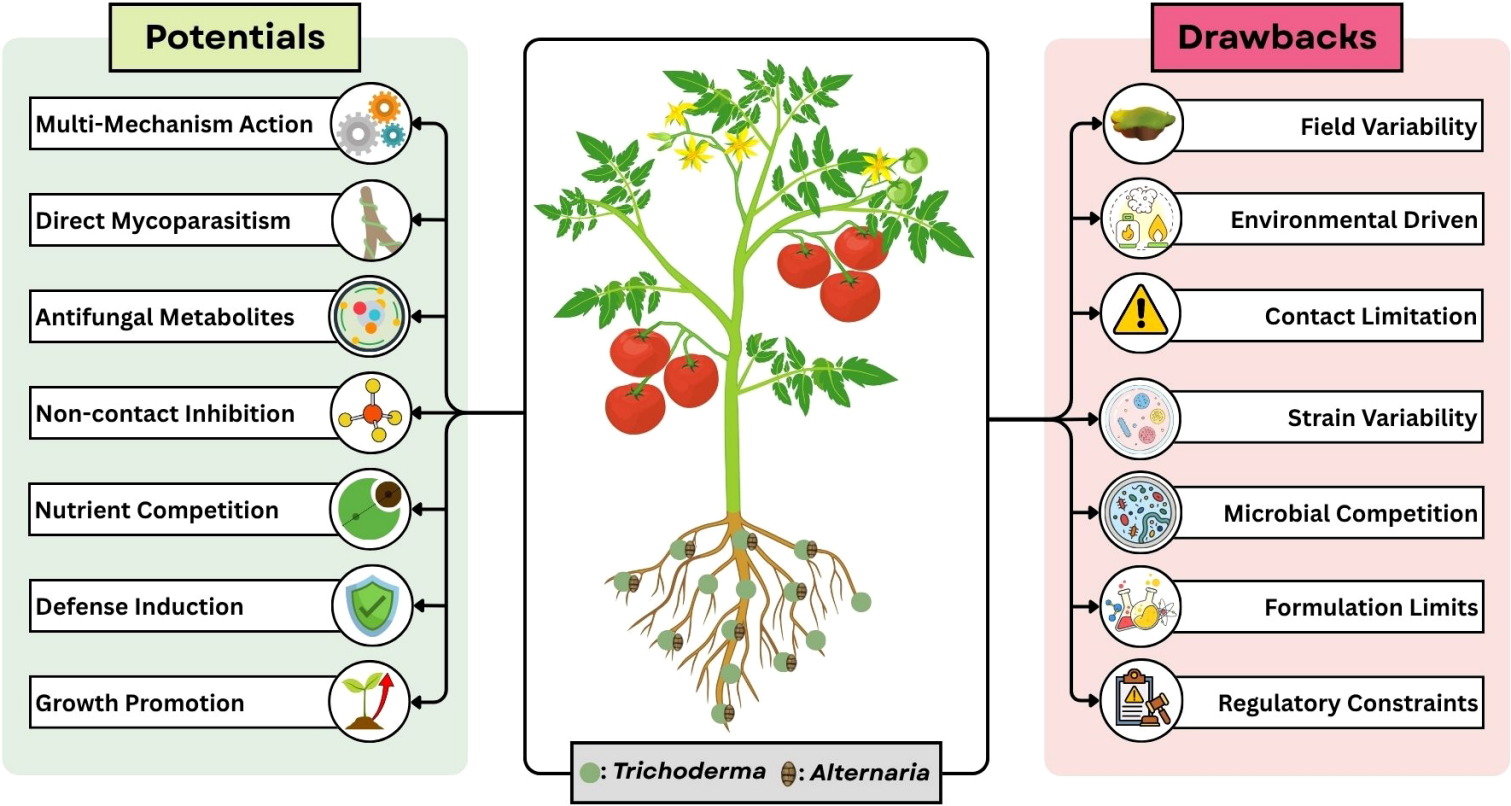
Summary of the potentials and drawbacks of *Trichoderma* in managing *Alternaria*-caused diseases in horticultural crops.

Another major limitation concerns formulation stability and shelf-life, as maintaining the viability and biological activity of *Trichoderma* spp. during storage and field application remains challenging, particularly under suboptimal environmental conditions (Rimkus et al., 2023; Løvschall et al., 2024; Arias-Chavarría et al., 2025). Furthermore, regulatory and commercialization constraints, including complex registration procedures, strict quality control requirements, and challenges related to large-scale production, may hinder the wider adoption of *Trichoderma*-based biocontrol products (Chen et al., 2025; Rezaee Danesh et al., 2025). Potential non-target effects also warrant careful consideration, as the introduction of microbial biocontrol agents may influence indigenous microbial communities or alter ecological interactions within the soil microbiome (Shahriar et al., 2022; Alukumbura et al., 2022; Jaramillo-López et al., 2024; Jefferson et al., 2026c). Although *Trichoderma* spp. are generally regarded as environmentally safe, the long-term ecological consequences of their large-scale application remain insufficiently understood, highlighting the need for further investigation to ensure environmental sustainability, as illustrated in **Figure 3** (Guzmán-Guzmán et al., 2025; Moussa and Iasur Kruh, 2025; Win et al., 2026).

## 7 Future perspectives

Recent advances in genomic and metabolomic technologies are opening new opportunities to better understand the molecular mechanisms underlying *Trichoderma*-mediated biocontrol. High-throughput sequencing and functional genomics enable the identification of specific genes and metabolic pathways involved in antagonism, plant interaction, and stress tolerance. These tools provide valuable insights for future strain selection and improvement (Zhang et al., 2025; Sun et al., 2025; Vargas-Gasca et al., 2026). Furthermore, molecular approaches such as gene-editing technologies, including CRISPR/Cas systems, offer promising strategies for developing improved *Trichoderma* strains with enhanced biocontrol efficacy and superior environmental adaptability (Wang et al., 2022c; Zhang et al., 2024c).

Another promising direction is the application of multi-strain consortia to enhance the stability and effectiveness of biological control (Joshi et al., 2023; Nunes et al., 2024; Kredics et al., 2024). The combination of different beneficial microorganisms can promote synergistic interactions that enhance disease suppression and broaden the spectrum of activity across diverse environmental conditions (Minchev et al., 2021; Nunes et al., 2024; Kredics et al., 2024). This approach addresses the limitations of single-strain applications by creating more robust microbial communities capable of withstanding ecological fluctuations.

In the long term, integrating *Trichoderma* into sustainable horticultural disease management systems remains a primary goal. When combined with cultural practices, the use of resistant cultivars, and a reduced reliance on chemical inputs, *Trichoderma*-based strategies may support the development of more resilient and environmentally sustainable crop protection frameworks (Gutiérrez-Moreno et al., 2025; Yao et al., 2023). Future research should prioritize the transition from laboratory models to large-scale, integrated field systems to ensure that the potential of these beneficial fungi is fully realized in global horticultural production.

## 8 Conclusion

This review synthesizes current knowledge regarding the biological characteristics and antagonistic mechanisms of *Trichoderma* spp. in the management of *Alternaria*-associated diseases in horticultural crops. Evidence from laboratory, greenhouse, and field studies consistently demonstrates that *Trichoderma* spp. employ multiple complementary modes of action. These include mycoparasitism, antibiosis, nutrient and spatial competition, and the induction of plant systemic resistance. These mechanisms, combined with the capacity of *Trichoderma* spp. to colonize plant roots and interact with the rhizosphere microbiome, enable the effective suppression of *Alternaria* pathogens while simultaneously promoting plant growth and stress tolerance. The multifunctional nature of these interactions highlights the ecological versatility of *Trichoderma* and its capacity to contribute significantly to improved plant health and productivity.

Beyond direct pathogen suppression, the integration of *Trichoderma*-based biocontrol strategies offers a promising pathway toward reducing the reliance on chemical fungicides in horticultural production systems. By combining antagonistic activity with plant growth–promoting effects and enhanced stress resilience, *Trichoderma* spp. function not only as a biocontrol agent but also as a key component of sustainable crop management. The growing body of experimental and field-based evidence suggests that incorporating *Trichoderma* into integrated disease management programs can support environmentally responsible disease control while maintaining crop yield and quality. Such approaches align with global efforts to develop more resilient agricultural systems capable of meeting increasing food demands while minimizing environmental impacts.

Despite these promising attributes, several challenges remain that influence the consistency and large-scale adoption of *Trichoderma*-based biocontrol strategies. Variability in field performance, formulation stability, ecological interactions with indigenous microbiota, and regulatory constraints continue to limit broader implementation. Future research should therefore focus on improving strain selection, optimizing formulations and delivery systems, and integrating advanced genomic and metabolomic tools to better understand and enhance the functional traits of *Trichoderma*. Ultimately, the successful application of *Trichoderma* in horticultural disease management will depend on combining scientific innovation with practical agricultural strategies to ensure reliable, scalable, and environmentally sustainable plant protection.
